# Introducing One Health Advances: a new journal connecting the dots for global health

**DOI:** 10.1186/s44280-023-00011-1

**Published:** 2023-03-29

**Authors:** Jianzhong Shen, Stefan Schwarz

**Affiliations:** 1 National Key Laboratory of Veterinary Public Health Security, College of Veterinary Medicine, China Agricultural University, Beijing, 100193 China; 2 Department of Veterinary Medicine, Institute of Microbiology and Epizootics, Centre of Infection Medicine, Freie Universität Berlin, 14163 Berlin, Germany

We are excited to announce the publication of the inaugural issue of our new golden open access journal, *One Health Advances*. This journal is dedicated to high-quality original research articles and reviews in the field of One Health, a holistic approach to public health that recognizes the interdependence of animal, environmental, and human health. In the light of the recent outbreak of COVID-19, the importance of a One Health approach to research has never been more evident.

Our primary goal with *One Health Advances* is to provide a platform for the rapid dissemination of cutting-edge scientific knowledge on topics, such as antimicrobial resistance (AMR), animal-derived food safety, and animal diseases with a preference for zoonoses. We are committed to multidisciplinary and multifaceted perspectives, and are relying on the expertise from leading experts across the globe in fields, such as microbiology, virology, epidemiology, genomics, chemistry, environmental science, health policy, clinical laboratory, and diagnostics science.

The articles featured in the inaugural issue of *One Health Advances* represent a diverse and important set of topics. For example, Qijing Zhang et al. take a One Health approach to review the prevalence and spread of zoonotic and antimicrobial-resistant *Campylobacter*, offering recommendations for control and mitigation [1]. Weiwei Yang et al. discuss the rise of AMR in China based on data from China Antimicrobial Surveillance Network (CHINET), highlighting the crucial role of surveillance systems in curbing this trend [2]. Weiwei Li et al. analyze the resistance profiles of *Salmonella* strains in Chinese diarrhea patients to provide guidance for better clinical antimicrobial treatment [3]. The studies by Yang Bai et al. and Gang Liu et al. investigate risk factors and transmission of *Porphyromonas gulae* and *Leishmania*, respectively, which cause diseases in dogs, and provide insight into appropriate disease management strategies [4, 5]. This issue also includes significant space devoted to antimicrobial resistance profiles in pets, closely related to humans, with studies by Shizhen Ma et al. and others, providing crucial data on antimicrobial resistance dynamics in pets and recommendations for pet medical care and public health [6].

In addition to these articles, this issue offers valuable insight into the ongoing emergence of influenza A viruses, with Chris Ka Pun MOK et al. suggesting the need for enhanced surveillance of mink influenza viruses for early warning of potential pandemics [7]. Shaoyuan Tan et al. provide a new strategy for the development of a porcine reproductive and respiratory syndrome virus (PRRSV) vaccine [8], while studies by Ghulam Mujtaba Mari et al. and Weifang Gu et al. offer potential means for rapid, accurate, and reliable detection of antimicrobial residues in animal-derived foods and vegetables [9, 10].

We believe that these studies offer a glimpse into the breadth and scope of research that *One Health Advances* will publish. If any of these topics pique your interest, we encourage you to read the corresponding articles. We would like to extend our sincere thanks to all the authors who have contributed to this inaugural issue. We hope that the launch of *One Health Advances* marks the beginning of a beautiful journey connecting the various aspects of global health.
